# Complete mitochondrial genome of the jellyfish, *Nemopilema nomurai* (Cnidaria: Scyphozoa) and the phylogenetic relationship in the related species

**DOI:** 10.1080/23802359.2017.1298417

**Published:** 2017-03-23

**Authors:** Yantao Wang, Song Sun

**Affiliations:** a Key Laboratory of Marine Ecology and Environmental Sciences, Institute of Oceanology, Chinese Academy of Sciences, Qingdao, China;; b Laboratory for Marine Ecology and Environmental Science, Qingdao National Laboratory for Marine Science and Technology, Qingdao, China;; c Jiaozhou Bay Marine Ecosystem Research Station, Institute of Oceanology, Chinese Academy of Sciences, Qingdao, China

**Keywords:** *Nemopilema nomurai*, mitochondrial genome, next generation sequencing, phylogenetic relationship

## Abstract

The complete mitochondrial genome of giant jellyfish *Nemopilema nomurai* collected from East China Sea was determined by next-generation sequencing. The mitogenome is a circular molecule 17,024 bp in length, including 13 protein-coding genes (including Cox 1, Cox2, Atp 8, Atp 6, ND5, ND 6, ND3, ND41, ND1,ND4, Cytb), 6 tRNAs (tRNA-Trp, tRNA-Met, tRNA-Val, tRNA-Arg, tRNA-Glu, tRNA-Asn), 2 rRNA genes (small subunit RNA and large subunit RNA), and 1 putative control region. The phylogenetic tree in the related species showed that giant jellyfish is close to *Hydraoligaclis*.

The giant jellyfish *Nemopilema nomurai* (Cnidaria: Scyphozoa: Rhizostomeae: Stomolophidae) is endemic in the East Asian Marginal Seas, commonly encountered in the East Asian coastal waters (the Bohai Sea, the Yellow Sea, the East China Sea, and the Sea of Japan) and frequent blooms of this species have occurred during recent decades (Yoon et al. 2014; Sun et al. 2015).

To date, a few reports are available on complete mitochondrial genomes as in *Aurelia aurita* (Shao et al. 2006), *Aurelia* sp. nov. (Hwang et al. 2014a) and *Craspedacusta sowerbyi* (Zou et al. 2012) and *Chrysaora quinquecirrha* (Hwang et al. 2014b). It would be, therefore, important to analyze the complete mitochondrial genome to better understand the molecular phylogenetic relationship between Cnidaria species. In the present paper, we first report the complete mitochondrial DNA from *N. nomurai* in order to obtain basic genetic information within the genus *Stomolophidae*.

DNA from 1g bell tissue of a single specimen of *N. nomurai* weighing ∼10 kg collected from East China Sea (32.00N, 123.50E) was extracted by standard phenol–chloroform extraction method (Sambrook & Russell 2001) and part of the rest of the sample was preserved in −20 °C at KLMEES Institute of Oceanology with the voucher no. Nmit001.

DNA sequencing was prepared in paired end libraries, tagged and subjected to next generation sequencing (NGS) on the Illumina Next-Seq 500 system (Genotypic Technology Pvt. Ltd, Bengaluru, India). Quality check, raw read pre-processing, and *de novo* assembly was performed using CLC Genomics Workbench version 7.0.4 (Aarhus, Denmark). Characterization of *N. nomurai* mitogenome was carried out by comparing with a closely related fish mitogenome (Saitoh et al. 2011). A phylogenetic tree was constructed based on 10 representatives from Cichlidae family and one cyprinidae, complete mtDNA sequence.

The complete mitogenome of *N. nomurai* is 17,024 bp in length and the GenBank accession No. is KY454767. It consists of 13 protein-coding genes (including Cox 1, Cox2, Atp 8, Atp 6, ND5, ND 6, ND3, ND41, ND1,ND4, Cytb), 6 tRNAs (tRNA-Trp, tRNA-Met, tRNA-Val, tRNA-Arg, tRNA-Glu, tRNA-Asn), 2 rRNA genes (small subunit RNA and large subunit RNA), and 1 putative control region. All the genes showed complete stop codons using TAA and TAG. There is also slight anti-G bias (13.61% and 13.14%) on the 2nd and 3rd position of all the genes. The start codon of ND 3 and ND 6 are different from other species, such as *A. aurita* (Shao et al. 2006).

The mitochondrial genome base composition for 13 genes was 28.22% for A, 37.11% for T, 17.20% for G, and 17.47% for C. The A + T base composition (65.33%) was higher than G + C (34.67%), suggesting that the giant jellyfish has low G + C ratio in the mitochondrial genome. Phylogenetic relationship revealed NJ tree among four related species based on the complete mitochondrial genome downloaded from BCBI (Figure 1). The NJ phylogenetic tree showed that the giant jellyfish is close to *Hydra oligaclis* (GenBank No. EU237491) clustered in a separate branch, and was far related to other species.

**Figure 1.**
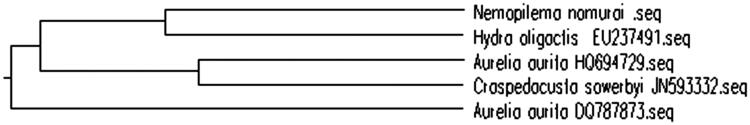
Phylogenetic relationship revealed by NJ tree.
